# Solar-Powered Switch of Antiferromagnetism/Ferromagnetism in Flexible Spintronics

**DOI:** 10.3390/nano13243158

**Published:** 2023-12-17

**Authors:** Chenying Wang, Yujing Du, Yifan Zhao, Zhexi He, Song Wang, Yaxin Zhang, Yuxuan Jiang, Yongjun Du, Jingen Wu, Zhuangde Jiang, Ming Liu

**Affiliations:** 1 State Key Laboratory for Manufacturing Systems Engineering, International Joint Laboratory for Micro/Nano Manufacturing and Measurement Technologies, School of Instrument Science and Technology, Xi’an Jiaotong University, Xi’an 710049, China; wangchenying@xjtu.edu.cn; 2 State Key Laboratory for Manufacturing Systems Engineering, Electronic Materials Research Laboratory, Key Laboratory of the Ministry of Education, School of Electronic Science and Engineering, Xi’an Jiaotong University, Xi’an 710049, China; duyujing@stu.xjtu.edu.cn (Y.D.); hezhexi96@stu.xjtu.edu.cn (Z.H.); 4122153052@stu.xjtu.edu.cn (Y.J.); ydu2019@stu.xjtu.edu.cn (Y.D.); jingen-wu@xjtu.edu.cn (J.W.); 3 State Key Laboratory for Manufacturing Systems Engineering, International Joint Laboratory for Micro/Nano Manufacturing and Measurement Technologies, School of Mechanical Engineering, Xi’an Jiaotong University, Xi’an 710049, China; wangsong2015@stu.xjtu.edu.cn (S.W.); zhangyaxin@stu.xjtu.edu.cn (Y.Z.); zdjiang@xjtu.edu.cn (Z.J.)

**Keywords:** flexible magnetoresistance sensor, photovoltaic spintronics, photo-induced electrons, SAF heterostructure

## Abstract

The flexible electronics have application prospects in many fields, including as wearable devices and in structural detection. Spintronics possess the merits of a fast response and high integration density, opening up possibilities for various applications. However, the integration of miniaturization on flexible substrates is impeded inevitably due to the high Joule heat from high current density (10^12^ A/m^2^). In this study, a prototype flexible spintronic with device antiferromagnetic/ferromagnetic heterojunctions is proposed. The interlayer coupling strength can be obviously altered by sunlight soaking via direct photo-induced electron doping. With the assistance of a small magnetic field (±125 Oe), the almost 180° flip of magnetization is realized. Furthermore, the magnetoresistance changes (15~29%) of flexible spintronics on fingers receiving light illumination are achieved successfully, exhibiting the wearable application potential. Our findings develop flexible spintronic sensors, expanding the vision for the novel generation of photovoltaic/spintronic devices.

## 1. Introduction

Flexibility endows electronics with portable, bendable, foldable, and lightweight features, making them increasingly interesting in both the marketing and scientific fields. In addition to these advantages, flexible spintronics possess the merits of energy efficiency and high integration density, opening up possibilities for various applications [1,2,3,4,5,6,7]. Nowadays, the study of spin flips under strain/stress effects fascinates researchers due to its intriguing physical properties. This research not only contributes to the development of flexible electronics but also enriches the potential applications of spintronics. For example, the giant magnetoresistance (GMR) effect, as synthetic antiferromagnetic (SAF) devices based on the Ruderman–Kittel–Kasuya–Yosida (RKKY) interaction, are considered essential alternatives with great promise for flexible spintronics [2,8,9,10,11,12]. However, achieving precise control of the RKKY interaction in this heterostructure is challenging due to limitations imposed by substrate roughness and surface nonplanarity [13,14]. Moreover, manipulating ferromagnetism with conformal magnetic tunnel junctions (MTJs) in flexible spintronic devices poses significant challenges.

Pure electron/spin current is currently considered to be a conventional method for manipulating ferromagnetism [15,16,17,18,19,20,21,22,23,24,25]. However, it suffers from a large interfacial current density (ranging from 10^11^ to 10^12^ A/m^2^) due to its low spin-current conversion efficiency. This leads to significant electrical heating issues [26,27,28]. Alternatively, other methods such as introducing a magnetic field (H field) or utilizing electrical measures to tune spin dynamics are also employed. Unfortunately, these methods have inherent flaws in spin manipulations. The traditional method of an H field for achieving spin flips is energy-inefficient and bulky. In addition, a high-operation electric field (E field) is required in ferroelectric (FE)/ferromagnetic (FM) heterostructures to electrically excite magnetoelectric (ME) coupling for tuning magnetism [29]. However, the use of FE/FM heterostructures results in integration challenges and clamping effect issues, and the input voltage triggers interfacial charge corrosive issues as well. These limitations restrict their applications in integrated circuit chips (ICs), necessitating the exploration of alternative modulated strategies.

Nowadays, the use of femtosecond or polarized lasers in spintronics has gained significant attention due to their ability to control ferromagnetism optically in an ultrafast manner [30,31,32,33,34,35,36,37]. However, the use of lasers in flexible and wearable applications is still hindered by the high costs associated with laser synthesis, laser-induced heating issues, and the complexity of the measurement process. In a recent study, we successfully demonstrated the use of photovoltaic/ferromagnetic heterostructures on rigid substrates to directly manipulate magnetic properties using natural light. This was achieved by exciting and migrating photo-induced electrons into the ferromagnetic layer, resulting in the alteration of spin properties [5,38,39,40,41,42]. The effectiveness of photo-induced electrons in manipulating spin dynamics at the nanoscale level has been proven. Therefore, solar-driven magnetic variation offers several favorable features, including a near-zero energy cost, a purely physical process without chemical contamination, and a low energy consumption, making it a more suitable solution for flexible spintronics.

In this study, we fabricated a flexible spintronic device on a polyethylene terephthalate (PET) substrate. We were able to achieve the shifting of anti-ferromagnetism (AFM) and FM states in a Co/Mo/Co/(PC71BM:PTB7-Th)/Pt heterostructure by photo-induced electron doping. The low current density of 10^2^ A/m^2^ was obtained using a photovoltaic active layer (PC71BM:PTB7-Th), as described in our previous study. The AFM/FM coupling was also found to be weakened under distinct curvatures (k = 1/3, 1/4, 1/5 mm^−1^) without sunlight irradiation, demonstrating the potential for dual manipulations of spin dynamics in flexible spintronic devices. Furthermore, we observed the stability and reversibility of the AFM/FM coupling in our flexible spintronic device after bending it to the corresponding curvatures 100 times. Importantly, we achieved almost 180° of photoelectron-driven switching of the AFM and FM states at a small H field (±125 Oe) in both the flat and bent states. We also observed all optical magnetization reversal, resulting in different magnetoresistance variations (15~29%) depending on the distinct sunlight intensities (0.8 suns~1.2 suns). These findings demonstrate the feasibility of integrating solar-driven flexible magnetoresistive sensors with a low current density and high durability into wearable electronic applications.

## 2. Results and Discussion

**Figure 1:**
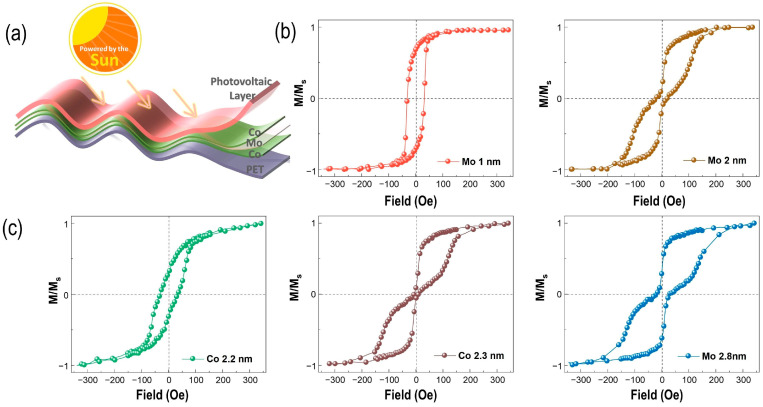
Schematics of the flexible photovoltaic spintronics. (**a**) The schematic of the multilayer structure fabricated on a flexible substrate. And, donor (PTB7-Th) and acceptor (PC71BM) compose the photo-active layer in heterojunctions. The magnetic trilayer (**b**), the magnetic hysteresis (M-H) loops of Co (2.5 nm)/Mo/Co (2.5 nm) full film with different $t_{Mo}$ in in-plane direction, and (**c**) the $t_{Co}$ dependence of M-H loops of Co/Mo (2.8 nm)/Co heterostructures in in-plane direction.

The schematic of a flexible PET/Co (2.5 nm)/Mo (2.8 nm)/Co (2.5 nm)/(PC71BM:PTB7-Th)/Pt (1 nm) heterostructure is illustrated in Figure 1a, to be used as a wearable electronic device. The two organic formulas of poly-[4,8-bis(5-(2-ethylhexyl)thiophen-2-yl) benzo [1,2-b:4,5-b′]dithiophene-co-3-fluorothieno [3,4-b] thiophene-2-carboxylate] (PTB7-Th) and [6,6]-phenyl C71 butyric acid methylester (PC71BM) are used as the electron donor and acceptor, respectively (Figure S1). These organic photovoltaic (OPV) molecules represent the most efficient donor–acceptor pair in the Fullerene system, in which the power conversion efficiency is over 10% [43,44]. Figure 1b,c reveals the thicknesses dependence of Co ($t_{Co}$) and Mo ($t_{Mo}$) with respect to exhibiting the RKKY interaction, showing periodical oscillations in FM and AFM coupling by altering the thickness of MTJs, respectively. And, a final thickness of Co ($t_{Co}$ = 2.5) nm and Mo ($t_{Mo}$ = 2.8 nm) can be obtained in the MTJ structure for exhibiting the AFM–FM coupling. Furthermore, the evolution of AFM–FM coupling in Co/Mo/Co heterostructures can be observed in Figure S2 in the Supplementary Materials.

**Figure 2:**
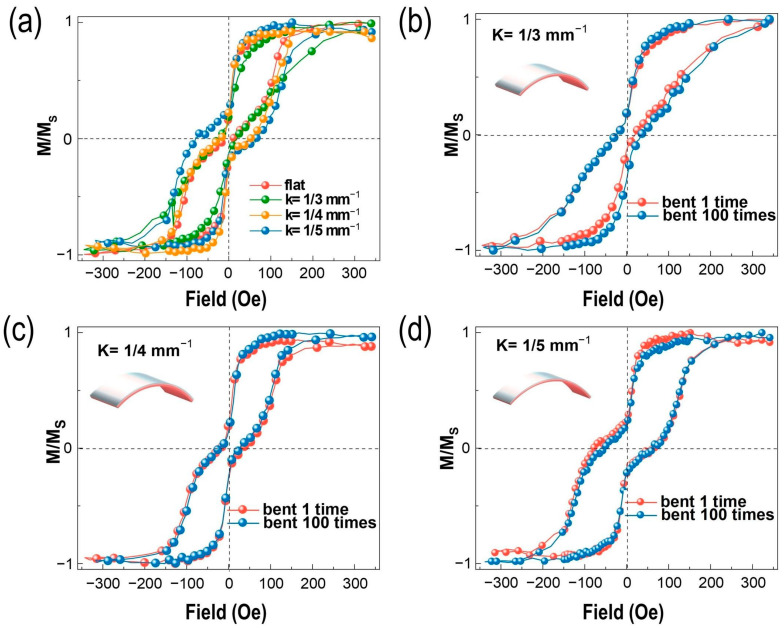
AFM–FM coupling in SAF multilayers under diverse bending states. (**a**) Different curvature measurement of Co2.5 nm/Mo 2.8 nm/Co 2.5 nm/PET SAT multilayers. The embedded graphs illustrate shape change and stress conditions for bend testing. (**b**–**d**) Flexibility measurement and repeat bending test for Co 2.5 nm/Mo 2.8 nm/Co 2.5 nm at ĸ = 1/3 mm^−1^, 1/4 mm^−1^, and 1/5 mm^−1^, respectively.

The normalized M-H loops of PET/Co/Mo/Co under diverse bending states are shown in Figure 2 for observing the variations in AFM coupling clearly. Figure 2a shows the hysteresis loops of the Co (2.5 nm)/Mo (2.8 nm)/Co (2.5 nm) heterostructure with/without curvatures of 1/3 mm^−1^, 1/4 mm^−1^, and 1/5 mm^−1^, respectively. The RKKY interaction can be varied by bending the film structure accordingly. The interfacial AFM coupling effect are weakened under the strain/stress effect in a convex bending shape. This demonstrates that the weakened AFM coupling originates from the bending cobalt film, where the magnetization can be re-orientated using the inverse magneto-strictive effect. This will affect the interlayer coupling strength due to the negative magnetostriction coefficient of cobalt. Then, the flexible SAF thin-film devices are bent under different curvatures (1/3 mm^−1^, 1/4 mm^−1^, and 1/5 mm^−1^) multiple times (100 times) to investigate the change in AFM coupling, shown in Figure 2b–d. The films exhibit excellent conformal performance and retain their magnetic coupling properties under every curvature (1/3 mm^−1^, 1/4 mm^−1^, and 1/5 mm^−1^). These bend radii are much smaller than ≈1 cm for matching the flexible electronic applications. Notably, the SAF films maintain their conformity with the PET substrate after being bent multiple times, illustrating the sufficient magnetic stability for the potential applications.

**Figure 3:**
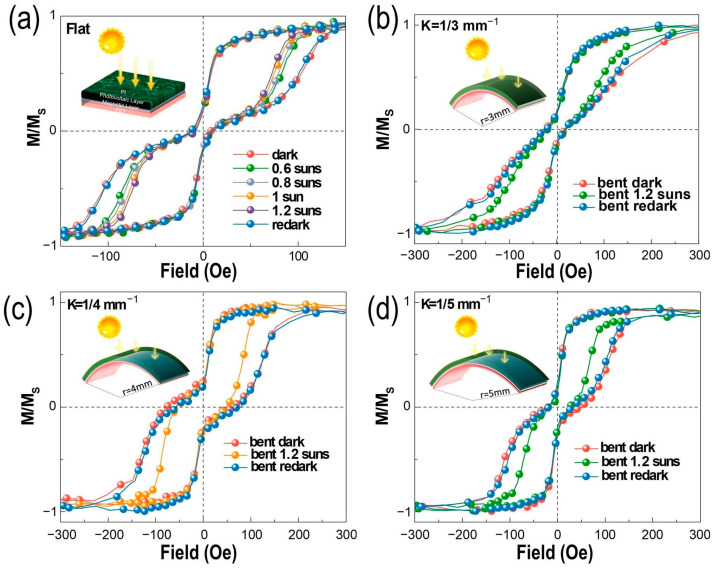
Deformation and light-soaking-induced changes in interlayer coupling strength in PET/Ta/Co/Mo/Co. (**a**) The VSM measurement under darkness, and 0.6-sun (60 mW/cm^2^), 0.8-sun (80 mW/cm^2^), 1-sun (100 mW/cm^2^), and 1.2-sun (120 mW/cm^2^) light conditions without bending. (**b**–**d**) The in situ light gating process at k = 1/3 mm^−1^, 1/4 mm^−1^, and 1/5 mm^−1^, showing the variations at darkness, 1.2 suns and re-dark conditions, respectively.

The in situ sunlight control of the RKKY interaction in the SAF heterostructure was characterized by the vibrating-sample magnetometer (VSM) measurement, illustrated in Figure 3. The bent films are fixed onto the nonmagnetic polytetrafluoroethylene (PTFE) with different curvatures (k = 0, 1/3, 1/4, and 1/5 mm^−1^). As is depicted in Figure 3a, the M-H loops shrunk gradually without any deformation in substrate as the light intensities increased from dark to 1.2 suns. This is attributed to the change in magnetic moment orientation from the anti-parallel state to the parallel state. These results demonstrate that AFM–FM coupling in the SAF layers can be altered by light illumination directly without the strain/stress effect. This consequence is consistent with the results of our previous study [41]. Generally, the photo-induced electrons (PIEs) are excited and migrated into Co/Mo/Co heterostructure to manipulate the interlayer exchange coupling (IEC) accordingly, leading to shifts from the AFM order to the FM order. However, the maximal shift in loop under 1.2-sun intensities of light illumination was observed. This indicates that the higher light intensity excites more PIEs, leading to a greater shift in RKKY interaction loops. On the other hand, the photovoltaic and strain effects are introduced simultaneously in the SAF heterojunctions to investigate the variations in AFM–FM coupling, as shown in Figure 3b–d. We can see that the synergic effect of strain and sunlight illumination cause a similar variation tendency in the RKKY interactions. The diminutions on AFM coupling can be observed under each curvature (1/3, 1/4, 1/5 mm^−1^) with light illumination (1.2 suns). Actually, the magnetic variation caused by sunlight was much smaller since the variation in AFM coupling under the bending state was not as obvious as that under a flat state. Even so, this indicates that sunlight can also weaken the AFM coupling under each bending state. However, large deformation affects the magnetic order, resulting in slight AFM coupling, as shown in Figure 3b. Meanwhile, the area of sunlight soaking is also diminished under a large curvature. Thereby, the minimal shifts in the RKKY interactions by PIE doping with deformation (k = 1/3 mm^−1^) is obtained. The shifts in the RKKY interactions with curvatures (1/4, 1/5 mm^−1^) under sunlight illumination are improved significantly due to the increase in area of light soaking, which promotes more PIE excitation (Figure 3c,d). Furthermore, the AFM coupling can be reversed when the light is removed. This reversibility originates from the recombination of PIEs without light illumination, exhibiting the potential application of such devices as flexible memory devices. These switchable AFM/FM states during sunlight soaking originates from the photo-induced electrons filling in the unoccupied band. This results in a rise in the Fermi energy to increasing the exchange interaction, which has been fully expounded in our previous study [41]. And, it is necessary to realize that the thermal effect during light illumination is excluded, as shown in Figure S3, further indicating that sunlight control of RKKY interactions is induced by PIE doping. 

**Figure 4:**
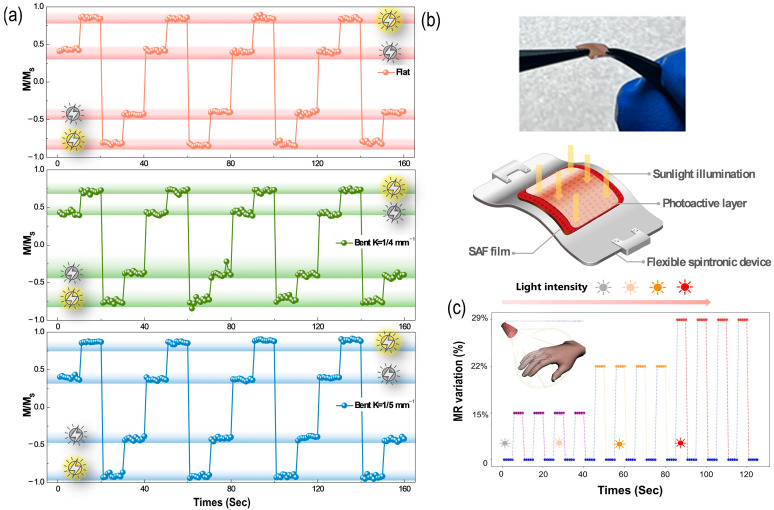
Solar-driven switch of magnetization in Co/Mo/Co heterojunctions by VSM measurement with applied H field (±125 Oe). (**a**) Switch of magnetization with bending curvature (k = 0, 1/4 mm^−1^, 1/5 mm^−1^) under light illumination (1.2 suns), corresponding to Figure 3a,c,d, respectively. The yellow sun symbol in the illustration indicates the light on, and the gray one represents the light off. (**b**) The practical and schematic images of flexible spintronic device. (**c**) The sunlight-induced MR variations in flexible spintronic device under different light intensities of illumination (from 0.8 suns to 1.2 suns) at curvature of 1/5 mm^−1^ on fingers.

Owing to the distinguished shifts in M-H loops under sunlight illumination with different curvatures (k = 0, 1/4, 1/5 mm^−1^), shown in Figure 3, the H field of ±125 Oe was applied to investigate the nearly 180° switch of magnetization in Co/Mo/Co by PIE doping, as shown in Figure 4a. We can see that the magnetization of the SAF heterostructure can be directly located at distinct positions with/without light. Similarly, the excellent reversibilities of magnetization were all observed before and after light illumination under bending curvatures (0, 1/4 and 1/5 mm^−1^). Furthermore, Figure 4a also demonstrates the obvious endurance and fast magneto–electric–optical response of flexible spintronics. Therefore, the flexible photovoltaic spintronic device was fabricated to explore its wearable applications. The reality and schematic images of flexible device are shown in Figure 4b, showcasing its miniaturization and flexibility. Then, the magnetoresistance (MR) variations in flexible spintronic device on fingers are demonstrated in Figure 4c. The device curvature of 1/5 mm^−1^ is maintained on fingers to detect the MR under different intensities of sunlight illumination (0.8 suns, 1 sun, 1.2 suns), resulting in magnetoresistance variations (15~29%). Our OPV/MTJ heterostructure device can sense the information at room temperature without spin current injection. This shows the potential feasibility on a GMR sensor for wearable magnetic memory and a light-intensity detector by establishing the photo–magneto coupling. This experimental application provides the possibility for solar-driven wearable spintronics, and opens up the approach for novel feasibility in the application of flexible spintronic devices.

## 3. Conclusions

In summary, the interlayer coupling on the flexible SAF sandwich heterostructure (Co/Mo/Co) was successfully modulated by sunlight illumination. The AFM/FM coupling could be weakened under distinct curvatures and PIE doping, demonstrating synergic manipulations of spin dynamics. On the other hand, the stability and reversibility of the AFM/FM coupling in the SAF structure were both achieved after bending 100 times. Notably, almost 180° of solar-driven switching of AFM and FM states at a small H field (±125 Oe) was also obtained. Finally, the distinct magnetoresistance variations (15~29%) of the device on worn fingers under light illumination were also achieved. The concept of a visible-light, tunable, magneto-resistive-response, flexible device was proposed. Our finding provides more possibilities for the next generation of wearable flexible devices and lays the foundation for the development of flexible spintronic devices. 

## 4. Experimental Section

### 4.1. Photovoltaic Layer Fabrication 

The PC_71_BM and PTB7-Th were purchased from 1-Material Chemscitech Inc., St-Laurent, QC, Canada. The weight ratio (1:1.5) of PTB7-Th and PC_71_BM was dissolved in the organic solvent (1,2-dichlorobenzene, ODCB) with 3% (*v*/*v*) 1,8-diiodooctane (DIO) for stirring overnight. The concentration of the mixed solution of PTB7-Th and PC_71_BM was 10 mg/mL. The photo-active layer (thickness of 70 nm) was fabricated using the spin-coating method at 1800 rpm onto Co/Mo/Co heterojunctions in the N_2_ atmosphere.

### 4.2. Device Fabrication

The device configuration of Co(25 Å)/Mo(28 Å)/Co(25 Å) was deposited onto the PET substrates using the magnetron sputtering method under Ar pressure of 5 mTorr. The base pressure before deposition was 3 × 10^−7^ Torr. The deposition rates of Co and Mo were 0.1 Å/s and 0.16 Å/s, respectively. Pt film was deposited onto the photovoltaic layer by magnetron sputtering as well.

### 4.3. In Situ Magnetic Property Measurement 

The magnetic hysteresis loops were recorded by vibrating-sample magnetometry at room temperature (7404 VSM system, Lake Shore Cryotronics, Inc., Columbus, OH, USA). The cylinder can be cut for achieving the desired curvature; then, the photovoltaic/ferromagnetic spintronic device is stuck onto the surface to maintain the curvature radius. After that, the sample under distinct curvatures can be located by VSM measurement with/without sunlight illumination for in situ testing. The test schematic is shown in Figure S4 in the Supplementary Materials. Flexible devices were irradiated using a solar simulator (PL-XQ500W Xenon lamp, Beijing Precise Technology Co., Ltd., Beijing, China) under AM1.5G (100 mW cm^−2^).

## Data Availability

Data are contained within the article and Supplementary Materials.

## Supplementary Materials

The following supporting information can be downloaded at: https://www.mdpi.com/article/10.3390/nano13243158/s1. Figure S1. The molecular structure of the donor (PTB7-Th) and acceptor (PC71BM) of the heterostructure junction. Figure S2. The evolution of AFM–FM coupling in Co/Mo/Co heterostructures with different thicknesses of Co and Mo, respectively. Figure S3. The RKKY interaction changes induced by thermal effect in light soaking process. Figure S4. The test schematic of in situ VSM measurement. The sample was fixed on a mold with a certain curvature to maintain bending state. The direction of the magnetic field was parallel to the sample (in plane), and the direction of visible light was perpendicular.
