# Supplementary material for: Solar-Powered Switch of Antiferromagnetism/Ferromagnetism in Flexible Spintronics
Source: Nanomaterials (Basel). 2023 Dec 17;13(24):3158. doi: 10.3390/nano13243158 (PMC10745959; doi:10.3390/nano13243158)
Supplement: Supplementary file 1 [file nanomaterials-13-03158-s001.zip › nanomaterials-2758522-supplementary.pdf]

Supporting Information

Solar-Powered Switch of Antiferromagnetism/Ferromagnetism in Flexible Spintronics

Chenying Wang [1,†], Yujing Du [2,†], Yifan Zhao [1,2,*], Zhexi He [2], Song Wang [3], Yaxin Zhang [3], Yuxuan Jiang [2],
Yongjun Du [2], Jingen Wu [2], Zhuangde Jiang [3] and Ming Liu [2,*]

[1] State Key Laboratory for Manufacturing Systems Engineering, International Joint Laboratory for Micro/Nano Manufacturing and Measurement Technologies, School of Instrument Science and Technology, Xi'an Jiaotong University, Xi'an 710049, China; wangchenying@xjtu.edu.cn

[2] State Key Laboratory for Manufacturing Systems Engineering, Electronic Materials Research Laboratory, Key Laboratory of the Ministry of Education, School of Electronic Science and Engineering, Xi'an Jiaotong University, Xi'an 710049, China; duyujing@stu.xjtu.edu.cn (Y.D.); hezhexi96@stu.xjtu.edu.cn (Z.H.); 4122153052@stu.xjtu.edu.cn (Y.J.); ydu2019@stu.xjtu.edu.cn (Y.D.); jingen-wu@xjtu.edu.cn (J.W.)

[3] State Key Laboratory for Manufacturing Systems Engineering, International Joint Laboratory for Micro/Nano Manufacturing and Measurement Technologies, School of Mechanical Engineering, Xi'an Jiaotong University, Xi'an 710049, China; wangsong2015@stu.xjtu.edu.cn (S.W.); zhangyaxin@stu.xjtu.edu.cn (Y.Z.); zdjiang@xjtu.edu.cn (Z.J.)

* Correspondence: zhaoyifan100@xjtu.edu.cn (Y.Z.); mingliu@xjtu.edu.cn (M.L.)

† These authors contributed equally to this work.

Keywords: flexible magnetoresistance sensor; photovoltaic spintronics; photo-induced electrons; SAF heterostructure

---

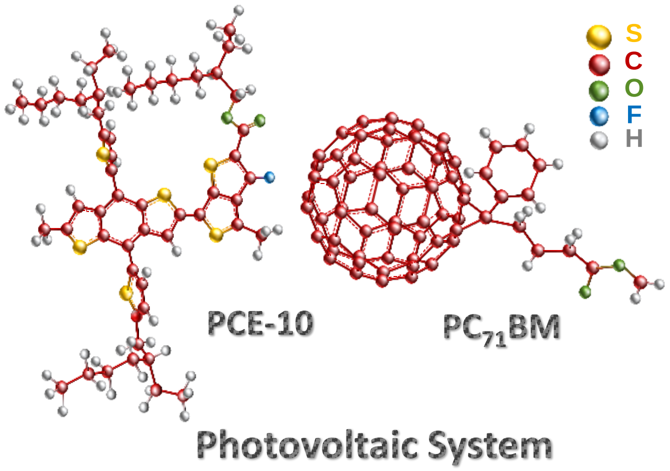

Figure S1. The molecular structure of the donor (PTB7-Th) and acceptor (PC71BM) of the heterostructure junction.

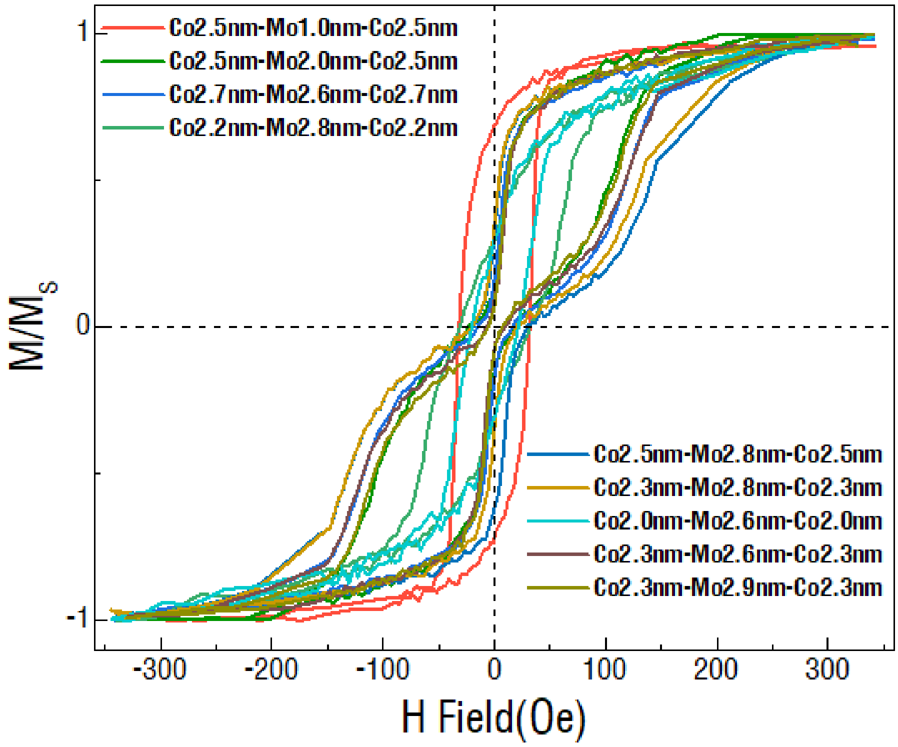

Figure S2. The evolution of AFM–FM coupling in Co/Mo/Co heterostructures with different thicknesses of Co and Mo, respectively.

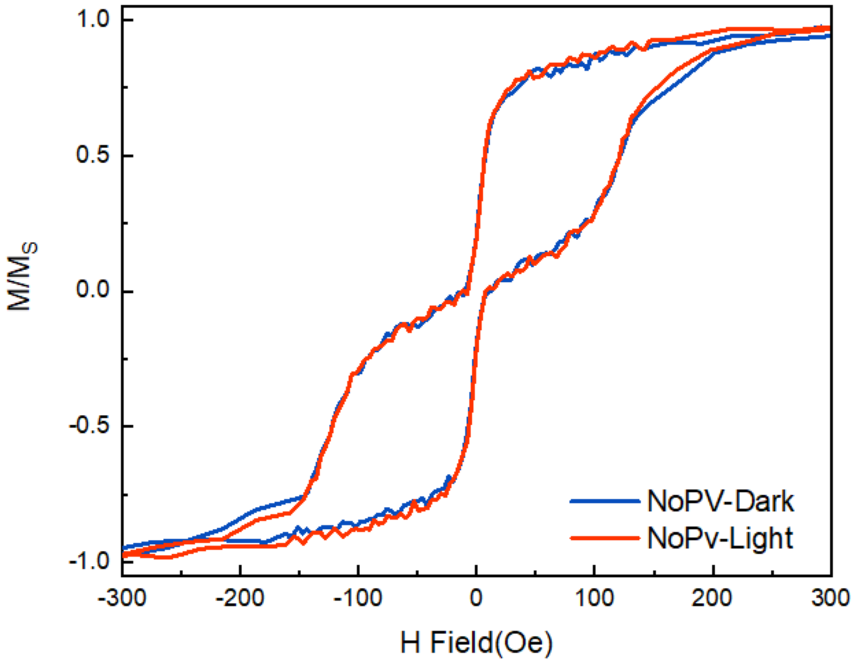

Figure S3. The RKKY interaction changes induced by thermal effect in light soaking process.

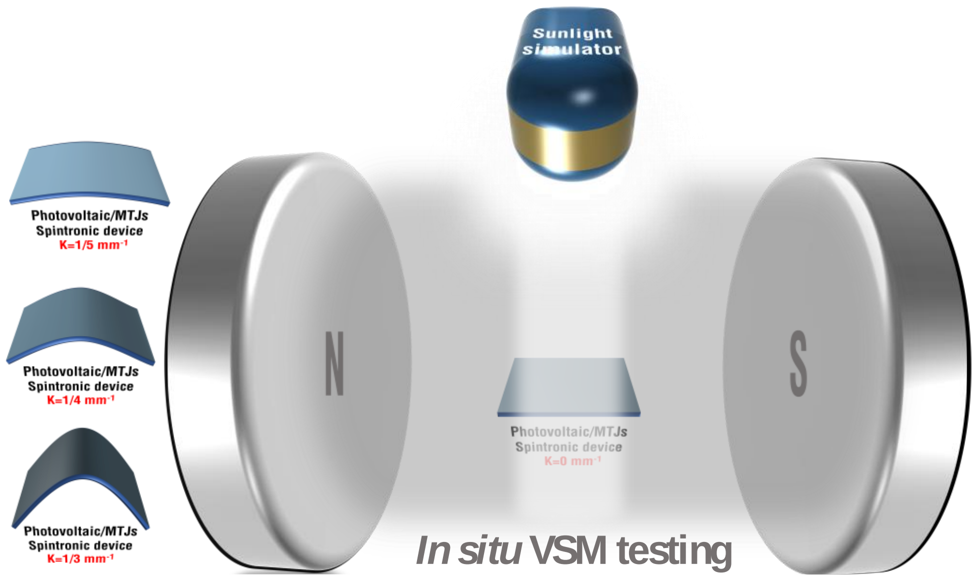

Figure S4. The test schematic of in situ VSM measurement. The sample was fixed on a mold with a certain curvature to maintain bending state. The direction of the magnetic field was parallel to the sample (in plane), and the direction of visible light was perpendicular.
